# Evaluation of the Protective Role of Vitamin E against ROS-Driven Lipid Oxidation in Model Cell Membranes

**DOI:** 10.3390/antiox13091135

**Published:** 2024-09-20

**Authors:** Dilara Kilicarslan You, Ahmed Fuwad, Ki Hyok Lee, Hyung Kyo Kim, Lifeng Kang, Sun Min Kim, Tae-Joon Jeon

**Affiliations:** 1 Department of Biological Sciences and Bioengineering, Inha University, 100 Inha-ro, Michuhol-gu, Incheon 22212, Republic of Korea; dilarakilicarslan@inha.edu; 2 Department of Mechanical Engineering, Inha University, 100 Inha-ro, Michuhol-gu, Incheon 22212, Republic of Korea; ahmed.fuwad@smme.nust.edu.pk; 3 Department of Biomedical Engineering and Sciences, School of Mechanical and Manufacturing Engineering, National University of Sciences and Technology (NUST), Islamabad 44000, Pakistan; 4 Department of Materials Research Center, Genpeau Corporation, Incheon 21990, Republic of Korea; khlee@genpeau.com (K.H.L.); hkkim@genpeau.com (H.K.K.); 5 School of Pharmacy, Faculty of Medicine and Health, University of Sydney, Pharmacy and Bank Building A15, Sydney, NSW 2006, Australia; lifeng.kang@sydney.edu.au; 6 Biohybrid Systems Research Center (BSRC), Inha University, 100 Inha-ro, Michuhol-gu, Incheon 22212, Republic of Korea; 7 Department of Biological Engineering, Inha University, 100 Inha-ro, Michuhol-gu, Incheon 22212, Republic of Korea

**Keywords:** lipid membranes, lipid oxidation, reactive oxygen species, hydrogen peroxide, antioxidants, vitamin E

## Abstract

Reactive oxygen species (ROS) are chemically reactive oxygen-containing compounds generated by various factors in the body. Antioxidants mitigate the damaging effects of ROS by playing a critical role in regulating redox balance and signaling. In this study, the interplay between reactive oxygen species (ROS) and antioxidants in the context of lipid dynamics were investigated. The interaction between hydrogen peroxide (H_2_O_2_) as an ROS and vitamin E (α-tocopherol) as an antioxidant was examined. Model membranes containing both saturated and unsaturated lipids served as experimental platforms to investigate the influence of H_2_O_2_ on phospholipid unsaturation and the role of antioxidants in this process. The results demonstrated that H_2_O_2_ has a negative effect on membrane stability and disrupts the lipid membrane structure, whereas the presence of antioxidants protects the lipid membrane from the detrimental effects of ROS. The model membranes used here are a useful tool for understanding ROS–antioxidant interactions at the molecular level in vitro.

## 1. Introduction

Reactive oxygen species (ROS) are generated from the incomplete reduction of oxygen during normal tissue metabolic processes, often stemming from oxygen consumption. ROS have been implicated in genetic instability and the initiation of tumorigenesis because of their destructive effects on DNA, proteins, and lipids [1]. Hydrogen peroxide (H_2_O_2_), an ROS, is relatively stable and plays a critical role in several metabolic pathways. Under normal and healthy conditions, the production and excretion of H_2_O_2_ is balanced. However, oxidative stress can disrupt this balance, leading to the excessive generation of H_2_O_2_ [2,3,4]. To maintain redox homeostasis, cells use antioxidant defense mechanisms to prevent the accumulation of excessive amounts of H_2_O_2_. Vitamin E plays an essential role in reproduction and is primarily recognized for its potent antioxidant properties [5]. It protects cells from oxidative damage by scavenging free radicals, thereby reducing the risk of oxidative stress-related conditions [6,7]. Several studies have shown that vitamin E has strong anti-proliferative, anti-survival, pro-apoptotic, and anti-angiogenic effects [5,8]. Even though the molecular basis of vitamin E remains unclear, vitamin E deficiency is physiologically associated with fertility and neuromuscular dysfunction [8,9].

The oxidative state of a cell depends on the balance between oxidant and antioxidant components. An imbalance leads to oxidative stress, cellular toxicity, and other pathological changes [10,11]. Disturbances in membrane properties, such as fluidity and permeability, caused by oxidation-induced changes can lead to the leakage of vital organelle components, leading to abnormal and dysfunctional protein aggregates [12,13]. Therefore, understanding the membrane behavior in relation to the interplay between oxidant and antioxidant elements and considering the level of lipid unsaturation is both important and complex.

Studies on ROS have focused on singlet oxygen (^1^O_2_) generated by photodynamic therapy. In a recent study, the damaging effects of photodynamic therapy-generated ROS were investigated using giant unilamellar vesicles (GUVs) in which ROS exposure induced membrane deformation [14]. Another study showed that the presence of a fluorogenic α-tocopherol analog, H_4_BPMHC, later protected the vesicles from deformation and surface expansion due to its antioxidant properties [15]. In addition, the influence of H_2_O_2_ on membrane permeation was investigated in 1,2-dioleoyl-sn-glycero-3-phosphocholine (DOPC) membranes containing different compositions of oxidized lipids, indicating the potential routes of H_2_O_2_ permeation [16]. Nevertheless, the existing literature on H_2_O_2_-related research remains limited. Moreover, the use of antioxidants as protective scavengers against hydrogen peroxide interactions is also limited [16,17,18].

Therefore, this study aimed to investigate the intricate interplay between vitamin E and lipid oxidation in model cell membranes, particularly in the induction of H_2_O_2_. To examine the impact of H_2_O_2_ and vitamin E on membranes, unsaturated and saturated phospholipids were employed to prepare a variety of model cell membranes. Following the addition of H_2_O_2_, the oxidative damage within membranes prepared by unsaturated lipids in comparison with the saturated lipid membranes were confirmed. However, the presence of vitamin E demonstrated a crucial effect on stabilizing the membrane by neutralizing free radicals due to its antioxidant capacity. This finding provides insight into the interaction between ROS and antioxidants and has the potential to aid in the development of drugs to combat diseases triggered by both H_2_O_2_ and ROS.

## 2. Materials and Methods

### 2.1. Materials

The following reagents: 1,2-dioleoyl-sn-glycero-3-phosphocholine (DOPC); 1-palmitoyl-2-oleoyl-glycero-3-phosphocholine (POPC); 1,2-dimyristoyl-sn-glycero-3-phosphocholine (DMPC); and 1,2-dioleoyl-sn-glycero-3-phosphoethanolamine-N-(7-nitro-2-1,3-benzoxadiazol-4-yl) (ammonium salt; 18:1 NBD PE) were purchased from Avanti Polar Lipids (Alabaster, AL, USA). Chloroform (Sigma-Aldrich, St. Louis, MO, USA); dimethyl sulfoxide (DMSO; Sigma-Aldrich, St. Louis, MO, USA); an Avanti mini extruder kit (Avanti Polar Lipids, Alabaster, AL, USA); a 10mm polyester drain disc extruder membrane filter (Cytiva, Amersham Place, UK); 100 nm nucleopore polycarbonate membrane filters (Cytiva, Amersham Place, UK); glucose (Sigma-Aldrich, St. Louis, MO, USA); sucrose (Sigma-Aldrich, St. Louis, MO, USA); 25 mm coverslips (SPL Lifesciences Co, Ltd., Gyeonggi-do, Korea); α-tocopherol (vitamin E; Sigma-Aldrich, St. Louis, MO, USA); hydrogen peroxide (H_2_O_2_; Daejung Chem, Gyeonggi-do, Korea); bovine serum albumin (BSA; Sigma-Aldrich, St. Louis, MO, USA); an RF-5301PC spectrophotometer (Shimadzu Europe, Institute, Arlington, TX, USA); an ELS-Z Zeta Potential Analyzer (Otsuka Electronics Co., Ltd., Seongnam-si, Korea); a polytetrafluoroethylene film (PTFE film; Good Fellow, Huntingdon, UK); a spark generator (DAEDALON, Salem, MA, USA); a Digidata 1440A instrument and Axopatch 200B patch clamp (Molecular Devices, Sunnyvale, CA, USA); a digital microscope (Digital Blue, QX5, Marietta, GA, USA); Clampfit 11 software (Molecular Devices, Sunnyvale, CA, USA); Clampex 11 software (Molecular Devices, Sunnyvale, CA, USA); ethylenediaminetetraacetic acid (EDTA; Sigma-Aldrich, St. Louis, MO, USA); potassium chloride (KCl; Junsei Chemical Co., Ltd., Tokyo, Japan); 4-(2-hydroxyethyl)-1-piperazineethanesulfonic acid (HEPES; Alfa Aesar, Ward Hill, MA, USA); and a Ag/AgCl electrode (Alfa Aesar, Ward Hill, MA, USA) were purchased. Fourier transform infrared (FTIR) spectrometer (JASCO FT/IR-6600, Tokyo, Japan) was utilized.

### 2.2. Liposomes Preparation

The thin-film hydration method was used for liposome preparation [19,20]. Liposomes were prepared using DOPC, POPC, and DMPC phospholipids separately, with a total lipid concentration of 5 mM in each vial. Chloroform was evaporated under a gentle stream of argon gas, and the vial was then placed in a vacuum for 2–3 h to remove the residual solvent. After confirming the formation of a thin film within the vials, an appropriate quantity of distilled water (Millipore, Billerica, MA, USA) was used to rehydrate the lipids. The solutions were extruded 21 times through 100 nm nucleopore polycarbonate membrane filters using a mini-extruder kit to obtain liposomes. These were then used for further analysis.

### 2.3. Fluorescence Analysis

Five millimolar of each lipid solution prepared in Section 2.2 was labelled with 1 mol% NBD-PE. To ensure the integrity of the lipid membranes, 500 μM H_2_O_2_ and vitamin E were added to each lipid solution. Vitamin E was dissolved in DMSO to achieve a final concentration of 2 mM, with the vitamin E/DMSO solution constituting 8% of the total volume in a final DMSO/water ratio of 0.08 (8%). This carefully controlled solvent environment is crucial for maintaining lipid stability during the experiments. The fluorescence intensity was measured using an RF-5301PC spectrophotometer at an Ex/Em = 488/600 nm.

### 2.4. Size and Zeta Potential Measurements

Optimized concentrations of H_2_O_2_ and vitamin E were used for size and zeta potential measurements. Following the procedure outlined in Section 2.2, the liposomes were prepared and extruded 21 times through a 100 nm polycarbonate membrane filter. Size and zeta potential analyses were performed prior to (control samples) and following treatment with H_2_O_2_ and vitamin E. The zeta potential and dynamic light scattering were measured using an ELS-Z instrument.

### 2.5. GUV Preparation

GUVs were prepared using the hydrogel-assisted method [21,22]. Agarose with a low melting temperature was prepared at 70 °C. To cover the entire area, a small amount of agarose was added to one coverslip, which was then covered with another. The coverslips were separated and allowed to dry at room temperature. Because NBD-PE lipids are photosensitive, they were also used to create GUVs. To prepare the GUVs, 10 μL of a 1 mM lipid solution of DOPC, POPC, and DMPC were added separately, along with 1 mol% of NBD-PE in a dropwise manner onto the coverslips. The lipid solution was then spread on one side of the coverslip and applied to the entire surface using a cell spreader. The coverslip was sandwiched between O-rings within the 3D-printed cell chambers. To hydrate the lipid layer, a 50 mM sucrose solution was added to the chamber and incubated for 30 min to form GUVs. Once the GUVs were harvested, they were mixed with 52 mM glucose and incubated for 10–20 min before imaging. The 96-well plate was coated with 3% BSA and incubated for 30 min, followed by two rinses with distilled water. The GUVs were then visualized using an inverted fluorescence microscope, and the images were processed using ImageJ 1.54f software (NIH, Bethesda, MD, USA).

### 2.6. Fourier Transform Infrared (FTIR) Analysis

FTIR was used to confirm the presence of functional groups following H_2_O_2_ and vitamin E treatment of the liposomes. The liposome samples were freeze-dried prior to the IR analysis to avoid any potential effects of water molecules. The freeze-dried samples were combined with potassium bromide (KBr) powder and compressed to produce thin-crystal films. The film was placed in an FTIR spectrometer, and the data were measured in transmission mode. To detect changes in the absorbance spectra, the samples were examined before and after the addition of H_2_O_2_ and vitamin E.

A spectral subtraction analysis was performed using FTIR to identify changes in the lipid membranes after treatment with H_2_O_2_ and vitamin E. Infrared spectra were collected for lipid samples before and after treatment, focusing on the methyl (1370–1380 cm^−1^) and methylene (2845–2865 cm^−1^) bands, which indicate changes in lipid chain conformation and fluidity. Baseline correction and normalization were performed on each spectrum based on an internal standard band to ensure comparability.

### 2.7. Electrophysiological Measurements

A black lipid membrane (BLM) was fabricated using the painting method [23,24,25,26]. In 10 µm thick PTFE films, a spark generator was employed to create apertures. Apertures ranging from 160 µm in diameter were generated on one film. DOPC, POPC, and DMPC were dissolved in n-decane to form a 30 mg/mL lipid solution. The lipid solution was applied to both sides of the PTFE film aperture using the painting technique. This was followed by drying for 30 min. Next, a buffer solution (1 M KCl, 10 mM HEPES, and 1 mM EDTA; pH 7.0) was poured into both sides of the chamber at a total volume of 2 mL. The aperture was monitored using a digital microscope, and a bilayer was formed by painting lipid drops onto the aperture. To determine the presence of a bilayer, a triangular voltage signal was applied (±50 mV/20 ms). After the formation of the bilayer, 500 µM H_2_O_2_ and 2 mM vitamin E were added to the chamber to carry out the experiment.

### 2.8. Data Acquisition and Analysis

A Digidata 1440A instrument and an Axopatch 200 B patch clamp were connected to a thin Ag/AgCl electrode for experimentation. At a sampling rate of 100 kHz, data were collected using Clampfit 11 software and analyzed using Clampex 11 software. The conductivity difference was calculated from the triangular wave voltage and current variances, according to the following equation: [25]

(1) $$I\left(t\right)=C\times \frac{dV}{dt}$$

(2) $$C=\frac{\varepsilon_{0}\times \varepsilon_{S}\times A}{d}$$

where *C* is the capacitance, I is the current, *V* is the peak-to-peak voltage applied to the membranes, *ε*_0_ is the permittivity of free space (8.85 × 10 F/m), and *ε_s_* is the dielectric constant of the lipid. The dielectric constants of DOPC, POPC, and DMPC were 2.4, 2.8, and 2, respectively [27,28,29]. The area of the membrane is represented by *A*, and the bilayer thickness is denoted by *d*. The area of the membrane was determined using microscopic images of the BLM, and measurements were conducted using ImageJ software.

## 3. Results and Discussion

### 3.1. Characterization of Liposomes in the Presence of ROS and Vitamin E

#### 3.1.1. Size and Zeta Potential Measurements

**Figure 1:**
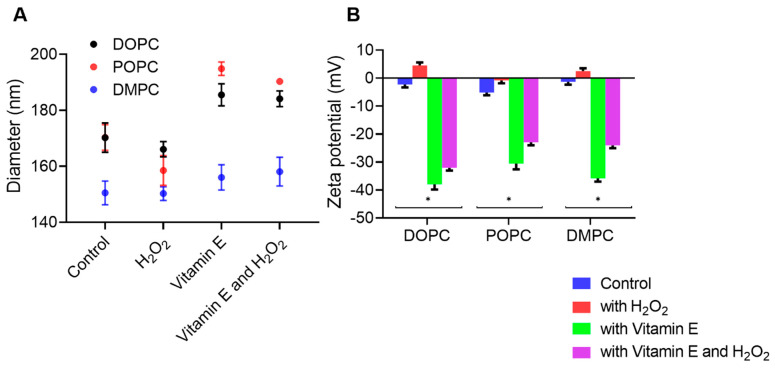
(**A**) Size distribution of DOPC, POPC, and DMPC liposomes with and without vitamin E and H_2_O_2_. (**B**) Zeta potential analysis of the DOPC, POPC, and DMPC liposomes interaction with and without vitamin E and H_2_O_2_. DOPC: 1,2-dioleoyl-sn-glycero-3-phosphocholine; POPC: 1-pal-mitoyl-2-oleoyl-glycero-3-phosphocholine; DMPC: 1,2-dimyristoyl-sn-glycero-3-phosphocholine. Error bars represent the means ± SEM of *n* = 4. Statistical significance was determined using a two-tailed *t*-test, * *p* < 0.05, compared with the control group.

The effects of ROS and vitamin E on liposome size and zeta potential were investigated using a particle size analyzer. The incorporation of H_2_O_2_ resulted in a reduction in the size and diameter of unsaturated lipids such as DOPC and POPC because of their unsaturated nature [30], whereas the saturated lipid, DMPC, did not experience a reduction (Figure 1A). Unsaturated lipids have one or more double bonds between the fatty acid carbon atoms. These double bonds cause the lipid structure to bend, thereby loosely packing the entire lipid molecule [31,32]. Upon exposure to oxidative stress caused by H_2_O_2_, these double bonds are particularly susceptible to radical formation. H_2_O_2_ readily abstracts a hydrogen atom from the methylene group adjacent to the double bond, forming a lipid radical. This radical can undergo further reactions, including coupling with other radicals, leading to the formation of cross-linked structures. These new structures are more rigid and tightly packed due to the cross-linking, which reduces the overall size and flexibility of the lipid bilayer. Consequently, the membrane can undergo significant alterations in both size and shape, as the formation of these cross-linked structures leads to tighter packing and a decrease in vesicle size [33]. The size changes observed for DOPC were less pronounced than those observed for POPC. This difference may be attributed to the specific molecular arrangements and packing densities of these lipids. DOPC and POPC exhibit different degrees of unsaturation, resulting in different oxidative stress responses. DOPC, with its two oleic acid chains, may exhibit a distinct packing density and structural configuration, rendering it less susceptible to observable disruptions under the experimental conditions used in this study. The double bonds present in both chains of DOPC may permit the formation of more flexible and less tightly packed structures, potentially providing resilience against oxidative disruption. Oxidation results in alterations to the molecular geometry of lipids, notably the conversion of lipid shapes from cylindrical to more conical forms due to the incorporation of oxidation products. This transformation affects the lipid packing parameter, which in turn influences the curvature and overall stability of the lipid bilayers. These geometric changes are pivotal in understanding the observed reduction in vesicle size as they promote tighter packing and increased curvature, leading to smaller vesicles [14,34,35]. In contrast, DMPC remained unchanged when exposed to H_2_O_2_. Saturated lipids, which have no double bonds in their fatty acid chains, are structurally rigid, rendering them less susceptible to deformation under oxidative conditions. For all liposomes, the addition of vitamin E or both vitamin E and H_2_O_2_ resulted in an increase in diameter. This is due to the interaction between vitamin E and lipids, which causes changes in lipid packing and results in an increase in liposome size. This can be explained by the strong binding capacity of vitamin E to unsaturated lipids, whereas in DMPC, vitamin E was positioned at the center of the lipid bilayer and not at the lipid–water interface. This arrangement of DMPC also reduces oxidation and prevents significant size changes [36,37,38,39]. The increased liposome size observed after treatment with H_2_O_2_, and vitamin E may be attributed to the presence of excess vitamin E molecules. While neutralizing free radicals (H_2_O_2_), the remaining unreacted vitamin E molecules may integrate into the lipid bilayer. This integration could lead to size alterations in liposomes, similar to those observed in the presence of vitamin E alone [40].

Surface potentials of DOPC, POPC, and DMPC liposome control samples were −2.30, −5.15, and −1.31 mV, respectively. The zeta potential charge increased upon the addition of H_2_O_2_. This increase in the zeta potential can be attributed to H_2_O_2_ molecules, suggesting that a neutral molecule may penetrate or accumulate on the membrane surface, causing a change in the zeta potential [2]. Furthermore, the addition of vitamin E resulted in highly negative surface potentials, indicating strong repulsion and good stability. The mean values observed for DOPC, POPC, and DMPC membranes were −38, −31, and −36 mV, respectively. The observed surface potentials were potentially due to the affinity of vitamin E to position itself on the membrane surface and expose its negatively charged carboxyl groups. Liposomes are highly charged due to the presence of vitamin E, leading to high electrostatic repulsion [41,42]. The addition of vitamin E in the presence of H_2_O_2_ resulted in a slight increase in the surface potential compared with the condition with vitamin E alone. Specifically, surface potentials of −32 mV for DOPC, −23 mV for POPC, and −24 mV for DMPC membranes were observed (Figure 1B). This is because the antioxidant properties of vitamin E neutralizes the free radicals formed by H_2_O_2_. Therefore, there are fewer charges on the liposome surface, resulting in an increase in the zeta potential.

#### 3.1.2. Molecular Analysis of Liposomes

FTIR was used to comprehensively investigate the influence of H_2_O_2_ on liposomes. This analytical approach provides valuable insights into the effects of experimental variables on the key molecular features of DOPC, POPC, and DMPC, such as symmetric methylene, carbonyl, and asymmetric phosphate stretching, in the presence and absence of vitamin E and H_2_O_2_.

**Figure 2:**
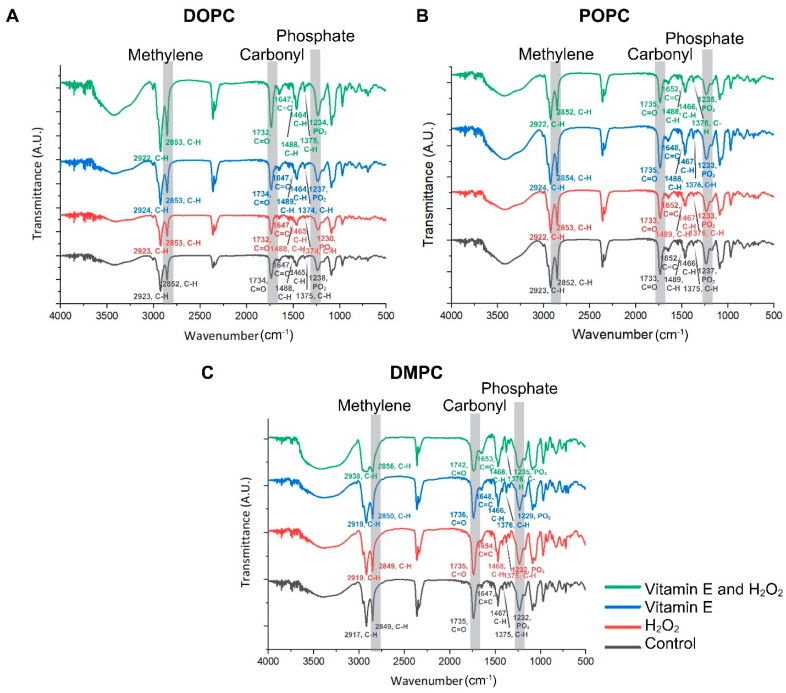
Structural characterization of the lipid membrane and vitamin E-H_2_O_2_ combination with lipid mixtures using (**A**) DOPC, (**B**) POPC, and (**C**) DMPC. FTIR: Fourier transform infrared spectroscopy, DOPC: 1,2-dioleoyl-sn-glycero-3-phosphocholine; POPC: 1-palmitoyl-2-oleoyl-glycero-3-phosphocholine; DMPC: 1,2-dimyristoyl-sn-glycero-3-phosphocholine.

**Figure 3:**
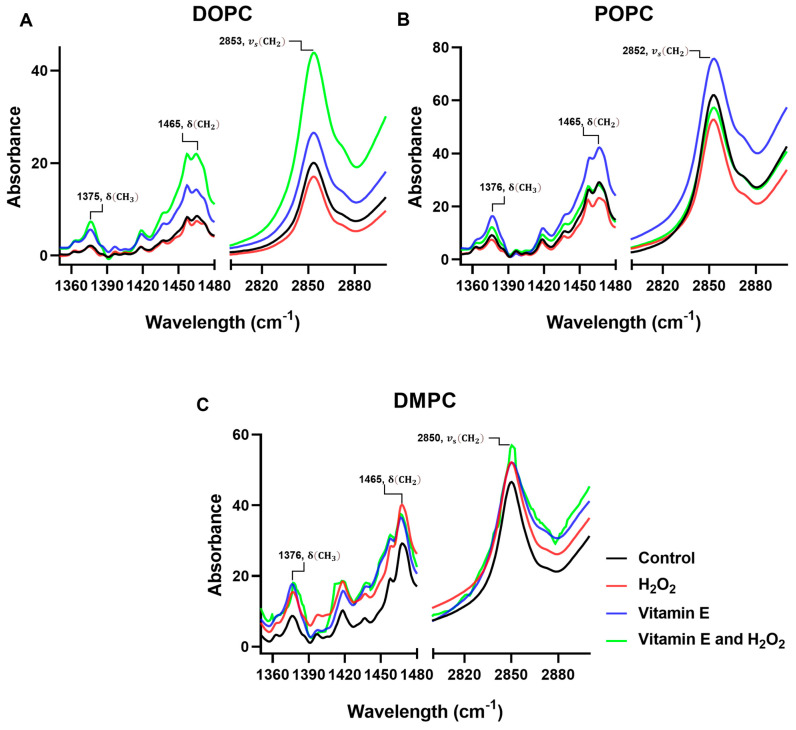
The results of the spectral subtraction analysis of the methyl and methylene bands of the lipid membrane and vitamin E-H_2_O_2_ combination with lipid mixtures using (**A**) DOPC, (**B**) POPC, and (**C**) DMPC. FTIR: Fourier transform infrared spectroscopy, DOPC: 1,2-dioleoyl-sn-glycero-3-phosphocholine; POPC: 1-palmitoyl-2-oleoyl-glycero-3-phosphocholine; DMPC: 1,2-dimyristoyl-sn-glycero-3-phosphocholine.

In comparison with the control groups of DOPC, POPC, and DMPC (Figure 2A–C and Figure 3), the addition of H_2_O_2_, vitamin E, or both did not result in any major changes in the observed frequencies (2852–2854 cm^−1^) for the methylene symmetric stretches of unsaturated DOPC and POPC in the FTIR spectrum, even though the reference points are 2845–2865 cm^−1^. The data indicated a slight decrease in DOPC and POPC membranes. The observed decrease in methylene band intensity suggests a reduction in lipid acyl chain order, which is consistent with the notion that membrane fluidity is increased upon treatment with H_2_O_2_ (Figure 3A,B). The observed frequencies were 2849–2856 cm^−1^ for saturated DMPC. The relationship between the variations in the frequency of methylene stretching in lipid membranes and the prevalence of disordered conformations in lipid chains are well established (Figure 3A–C). An increase in the methylene stretching frequency has been associated with increased levels of chain disorder, a phenomenon observed predominantly in saturated lipids [43,44,45]. In the context of DMPC, which is a saturated lipid, this could be indicative of a tighter packing of the lipid tails following treatment with H_2_O_2_ and vitamin E (Figure 3C). Carbonyl and phosphate groups were also identified in the FTIR spectra at 1742–1732 and 1238–1229 cm^−1^, respectively (Figure 2). For DOPC, the peak of carbonyl stretching shifted slightly from 1734 to 1732 cm^−1^ in the presence of H_2_O_2_ (Figure 2A). Similar shifts in the case of other phospholipids were attributed to variations in the number of carbonyl groups that were hydrogen-bonded (to either water or another local hydrogen-bonding donor, in this case, the alcoholic group on vitamin E), as carbonyl stretching includes components from both the tightly packed hydrogen-bonding and non-hydrogen-bonding bands [46]. The vitamin E hydroxyl group near the lipid–water interface is likely to interfere with the hydrogen bonding of DOPC and POPC with water by modifying the lipid interface distance and/or by competing for hydrogen bonding with water molecules at the interface with the carbonyl [47].

The bands between 1647 and 1653 cm^−1^ represent double carbon bond stretching vibrations. For both DOPC and POPC, the peak became weaker after the addition of ROS. However, the peak appeared to be stronger after the addition of vitamin E, either alone or in combination with H_2_O_2_. ROS affect double bonds, causing them to diminish or disappear, whereas vitamin E promotes membrane stability through its positioning on the membrane and antioxidant properties [48,49]. The double-bond region cannot undergo significant changes because of the saturated nature of DMPC, which contains only single bonds and no double bonds. As with the carbon double bond response on DOPC and POPC, the band located at 1488–1489 cm^−1^ showed a shoulder-like peak that decreased after ROS addition, while DMPC showed no meaningful change.

ROS can also affect the carbon–hydrogen (C-H) bonds, as observed near the 1465 and 1375 peaks [50,51], with methyl’s range (1370–1380 cm^−1^) serving as reference points (Figure 3). In DOPC and POPC, ROS caused a reduction in the C-H bonds, which was prevented by vitamin E. A reduction in the strength of the methyl band indicates that oxidative stress might directly impact the methyl groups or cause broader structural alterations in the lipid molecules, rendering these groups as less accessible or modified. It has been noted that an increase in the peak intensity of the methyl band suggests a stabilizing effect of vitamin E on the membrane. This effect likely results from the antioxidant capacity of vitamin E, which serves to maintain or restore the normal structural order of the membrane, thereby making the methyl groups more prominent or restoring them to their native state (Figure 3A,B). An increase in the intensity of the methyl bands near the 1376 and 1465 peaks was observed in DMPC. The enhanced methyl band intensity could indicate increased exposure or availability of the methyl groups in the lipid tails, potentially occurring as the lipid molecules reorient themselves in response to oxidative stress. This reorientation may serve as a mechanism to maintain membrane integrity or fluidity under conditions that are unfavorable for the organization of the lipid bilayer (Figure 3C) [39].

Asymmetric phosphate stretching in the control groups of DOPC and POPC were located at approximately 1237–1238 cm^−1^, while after addition of H_2_O_2_, vitamin E, and both vitamin E and H_2_O_2_, the peaks were at a slightly lower peak frequency (1230–1237 cm^−1^; Figure 2A,B). There was no significant difference observed between the control group of DMPC and the presence of H_2_O_2_, and both phosphate stretching peaks were centered at 1232 cm^−1^. Conversely, the presence of vitamin E only and vitamin E and H_2_O_2_ combined parameters exhibited slight changes in the phosphate stretching peaks at 1229–1235 cm^−1^ (Figure 2C). The phosphate band is widely recognized to be very sensitive to the level of hydration and the presence of ions [52,53]. Therefore, changes in the phosphate stretching frequencies were expected in the presence of either vitamin E or H_2_O_2_. 

#### 3.1.3. Liposomal Stability Analysis

Fluorescence measurements were conducted on liposomes containing NBD-PE lipids labeled with a fluorophore to gain a deeper understanding of membrane instability resulting from H_2_O_2_ and vitamin E treatments [54]. For fluorescence experiments, liposomes composed of DOPC, POPC, and DMPC were prepared using NBD-PE lipids. The concentrations of H_2_O_2_ and vitamin E were carefully selected to prevent excessive membrane disruption. Specifically, 500 μM H_2_O_2_ and 2 mM vitamin E were used.

**Figure 4:**
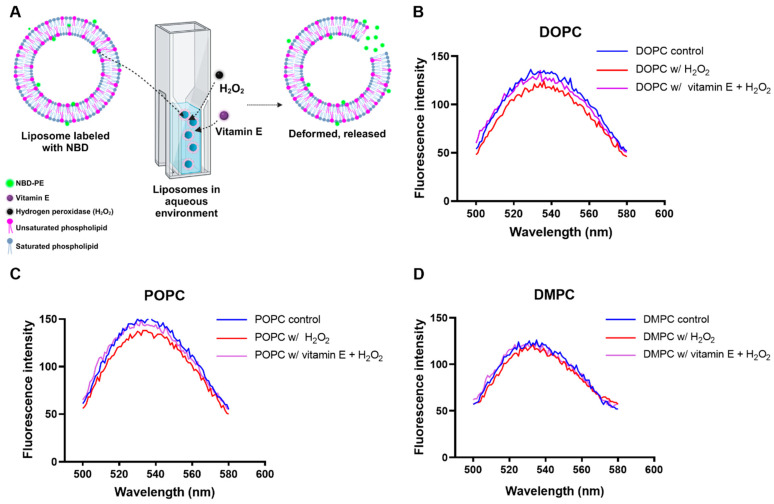
(**A**) Schematic representation of fluorescence intensity analysis of liposomes containing NBD-PE. Liposome fluorescence spectrum of (**B**) DOPC, (**C**) POPC, and (**D**) DMPC in different conditions. DOPC: 1,2-dioleoyl-sn-glycero-3-phosphocholine; POPC: 1-palmitoyl-2-oleoyl-glycero-3-phosphocholine; DMPC: 1,2-dimyristoyl-sn-glycero-3-phosphocholine; NBD-PE: 1,2-dioleoyl-sn-glycero-3-phosphoethanolamine-N-(7-nitro-2-1,3-benzoxadiazol-4-yl).

Following the addition of H_2_O_2_ to the cuvettes, a significant decrease in fluorescence intensity was observed for all DOPC and POPC liposomes, and a slightly lower decrease was observed for the DMPC liposomes (Figure 4). Loosening of the packing of liposome constituent lipids caused a disruption of the liposome structure due to lipid oxidation induced by H_2_O_2_. This led to a decrease in fluorescence intensity due to an increase in water–NBD fluorophore interactions, as hydrogen bond interactions between water and NBD atoms are another means of analyzing solvation. In addition, it is possible that some NBD molecules are located outside the liposomes. The introduction of H_2_O_2_ may result in additional enhancement of the quenching effect via interactions with water molecules and other quenching agents [18,31,54]. Vitamin E was added to the cuvettes to determine whether antioxidants could stabilize membrane disruption caused by free radicals [55]. The incorporation of vitamin E stabilizes the membrane by scavenging free radicals and reducing lipid oxidation. No visible changes in fluorescence were observed in comparison with the control experiments for all the samples. These results collectively suggest that the antioxidant capacity of vitamin E on lipids is due to its precise orientation in the membrane, allowing it to act as a lipid protector by scavenging reactive free radicals (Figure 4) [56,57].

### 3.2. Morphological Analysis of Model Cell Membranes

**Figure 5:**
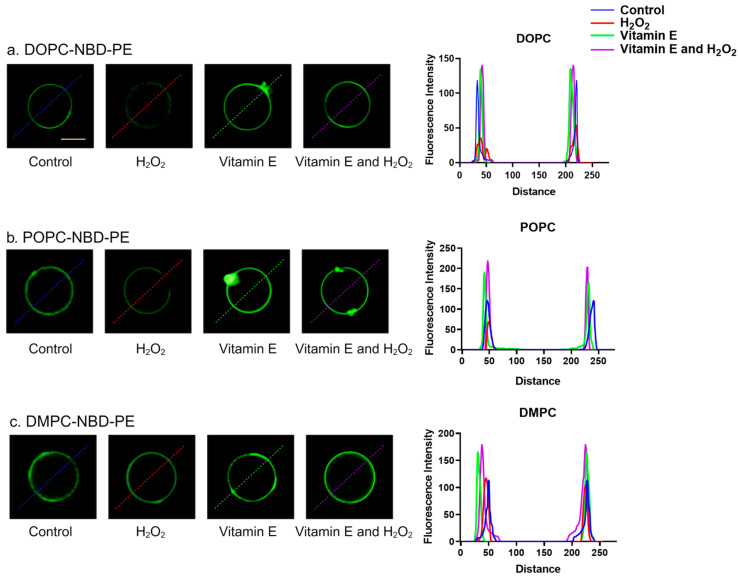
Fluorescence images of GUVs labeled with NBD (green). (**a**) DOPC-NBD, (**b**) POPC-NBD-PE, and (**c**) DMPC-NBD-PE GUVs and fluorescence intensity measurements. Fluorescence intensity was measured using ImageJ software along the colored dashed lines (**a**–**c**) for the three lipid groups. GUVs: giant unilamellar vesicles; DOPC: 1,2-dioleoyl-sn-glycero-3-phosphocholine; POPC: 1-palmitoyl-2-oleoyl-glycero-3-phosphocholine; DMPC: 1,2-dimyristoyl-sn-glycero-3-phosphocholine; NBD-PE: 1,2-dioleoyl-sn-glycero-3-phosphoethanolamine-N-(7-nitro-2-1,3-benzoxadiazol-4-yl). Scale bars = 20 µm.

Fluorescence microscopy was used to visualize the lipid bilayer and examine the interactions between H_2_O_2_ and vitamin E. The GUVs were generated using a hydrogel-assisted technique [21], incorporating 1 mol% of NBD-lipid (green; Figure 5). Spherical GUVs were observed in the microscopic images of all three liposome control samples (DOPC, POPC, and DMPC). However, upon the addition of H_2_O_2_, a decrease in fluorescence intensity was observed, owing to the unsaturated lipid structures of DOPC and POPC. H_2_O_2_ induces lipid oxidation, leading to membrane disruption. This disruption resulted in increased interactions between the water molecules and NBD, which quenched the fluorescence of NBD (Figure 5a,b). Because DMPC is a saturated lipid, the intensity changes after H_2_O_2_ addition were not similar to those of DOPC and POPC. Unlike DOPC and POPC, DMPC GUVs, which lack the unsaturated NBD-labelled control vesicles, did not show any morphological changes after the addition of H_2_O_2_. This confirms that the observed fluorescence dynamics were induced exclusively by the oxidation of unsaturated lipids by H_2_O_2_. In addition, significant membrane perturbation was observed in POPC GUVs, owing to lipid oxidation and surface area loss (Figure 5b). The absence of comparable perturbations in DOPC may be attributed to the differences in the lipid packing and unsaturation levels between DOPC and POPC. POPC exhibits greater susceptibility to disruption due to its distinctive structural characteristics and specific interactions with H_2_O_2_. In contrast, DOPC, despite its unsaturated state, may possess a distinct packing density or structural configuration, rendering it less prone to observable perturbations under the experimental conditions examined [14,34]. Following the addition of vitamin E to all control vesicles, membrane surface aggregation was observed mainly in DOPC and POPC (Figure 5a,b). This may be because vitamin E tends to interact with disordered membranes due to its high degree of unsaturation and binding affinity to the membrane interface [36]. With the lack of surface aggregation-like observations, DMPC may be attributed to the position of vitamin E on DMPC membranes, specifically at the center of the bilayers [39] (Figure 5c). In the presence of vitamin E and ROS, vitamin E, with its lipid-soluble domains, is readily positioned within the membrane, allowing it to interact with free radicals and halt lipid oxidation. This interaction effectively prevented a significant decrease in the signal intensity when vitamin E and H_2_O_2_ were present together [57].

### 3.3. Electrophysiological Analysis of Lipid Membranes

Electrophysiological studies were conducted using patch clamps to investigate the interactions between H_2_O_2_ and vitamin E on membranes, with the aim of understanding their influence on membrane stability and thickness. The BLM was used to study the physical and electrical changes exhibited by the lipid membrane in the presence or absence of H_2_O_2_ and vitamin E. The BLM functioned as an electrolyte reservoir formed across a small aperture, dividing the two chambers containing a buffer solution to mimic the aquatic environment. The electrical characteristics of the bilayer were analyzed by applying an electrical potential to the bilayer and measuring the resulting current [26,58]. The first step was conducted to confirm the BLM formation. All the procedures followed are described in Section 2. The membrane thickness was determined via the membrane area, followed by the calculation of the current using Equation (1) and (2) [25].

**Figure 6:**
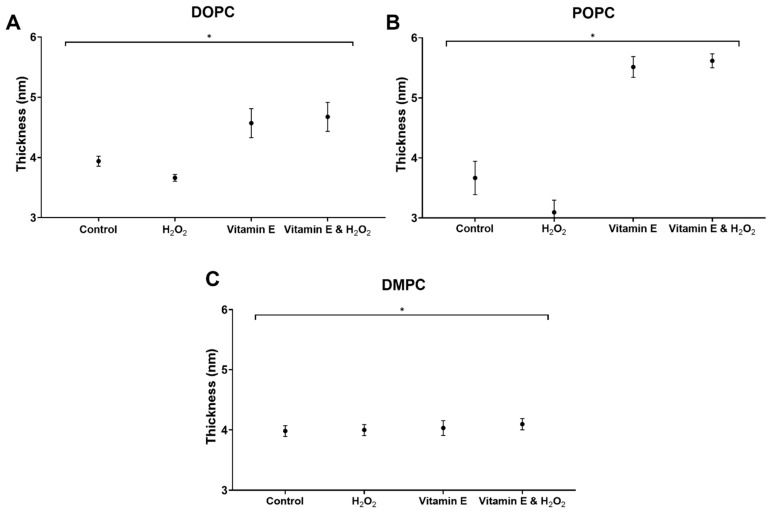
Electrical measurements of vitamin E and H_2_O_2_ flux on (**A**) DOPC, (**B**) POPC, and (**C**) DMPC BLMs. Lipid bilayer thickness was measured in the presence and absence of H_2_O_2_ and vitamin E with DOPC, POPC, and DMPC. DOPC: 1,2-dioleoyl-sn-glycero-3-phosphocholine; POPC: 1-palmitoyl-2-oleoyl-glycero-3-phosphocholine; DMPC: 1,2-dimyristoyl-sn-glycero-3-phosphocholine. Error bars represent the means ± SEM of *n* = 6. Statistical significance was determined using a two-tailed *t*-test, * *p* < 0.05, compared with the control samples.

The thickness of the BLM decreased in both the DOPC and POPC membranes when H_2_O_2_ was introduced into the chamber (Figure 6A,B). Unsaturated lipids are more susceptible to oxygen species than that of saturated lipids. Treatment with H_2_O_2_ or ROS, which causes lipid oxidation, resulted in the thinning of phospholipids on membranes, leading to a reduction in thickness [57,58]. ROS or H_2_O_2_ molecules interact with lipid molecules in the membrane, causing them to move apart from each other, resulting in a chemical disruption [55] that decreases the thickness of the membrane. This leads to a decrease in membrane stability and an increase in current flow. The thickness of the DMPC membranes remained mostly the same after H_2_O_2_ treatment, indicating that lipid oxidation did not affect the membranes (Figure 6C). After the application of vitamin E to the membranes, the DOPC and POPC membranes increased significantly and slightly in the DMPC membranes (Figure 6A–C). Regarding the affinity of vitamin E to unsaturated membranes in GUVs, a correlation was observed between the model membrane thickness results and this tendency. The thickness of the membrane increased because vitamin E has a high binding affinity for unsaturated lipids [36]. Vitamin E in DMPC is located at the core of the bilayer rather than at the lipid–water interface. This positioning prevents oxidation and detects noticeable changes in the thickness [39]. In the last step of the BLM experiment, H_2_O_2_ was applied after the application of vitamin E and then carefully mixed in a chamber. The membrane thickness was greater than that of the control group. The thickness of the DOPC and POPC membranes increased (Figure 6A,B). In contrast, the DMPC membrane showed results that were almost identical to those obtained under different conditions (Figure 6C). One explanation for the observed increase in membrane thickness following the application of H_2_O_2_ and vitamin E is that vitamin E neutralizes the free radicals present in the environment, thereby reducing their availability in the membrane bilayer [40]. A reduction in free radical levels may lead to membrane thickening.

## 4. Conclusions

In conclusion, the interactions between vitamin E and lipid oxidation, specifically induced by H_2_O_2_, using model cell membranes was investigated. Among these, unsaturated lipids, which are more prone to oxidative damage, are particularly susceptible to ROS, with H_2_O_2_ being a notable contributor to lipid oxidation. Remarkably, this investigation showed that, despite its stability, H_2_O_2_ disrupts membranes, highlighting its detrimental effects. Furthermore, vitamin E effectively neutralizes free radicals (H_2_O_2_), reducing their presence in the membrane bilayer and deactivating the reactions initiated by lipid oxidation. These findings enhance our understanding of how antioxidants such as vitamin E can protect lipid membranes from oxidative stress, providing a foundation for future studies on membrane stability and oxidative damage prevention.

## Data Availability

Data is contained within the article.

## Abbreviations

1,2-dioleoyl-sn-glycero-3-phosphocholine, DOPC; 1-palmitoyl-2-oleoyl-glycero-3-phosphocholine, POPC; 1,2-dimyristoyl-sn-glycero-3-phosphocholine, DMPC; 1,2-dioleoyl-sn-glycero-3-phosphoethanolamine-N-(7-nitro-2-1,3-benzoxadiazol-4-yl), NBD-PE; black lipid membrane, BLM; giant unilamellar vesicles, GUVs; hydrogen peroxide, H_2_O_2_; reactive oxygen species, ROS.
